# Retinoic Acid Signaling Is Required for Dendritic Cell Maturation and the Induction of T Cell Immunity

**DOI:** 10.4049/immunohorizons.2300022

**Published:** 2023-06-21

**Authors:** Mohammad Farazuddin, Nicholas Ludka, Leon Friesen, Jeffrey J. Landers, Jessica J. O’Konek, Chang H. Kim, James R. Baker

**Affiliations:** * Department of Internal Medicine, University of Michigan Medical School, Ann Arbor, MI;; † Michigan Nanotechnology Institute for Medicine and Biological Sciences, University of Michigan Medical School, Ann Arbor, MI;; ‡ Mary H. Weiser Food Allergy Center, University of Michigan Medical School, Ann Arbor, MI; § Department of Pathology, University of Michigan Medical School, Ann Arbor, MI

## Abstract

Vitamin A and its biologically active metabolites, all-*trans* and 9-*cis* retinoic acid (RA), are thought to be important in generating and modulating immune function. However, RA modulates the function of many types of immune cells, and its specific role in dendritic cell (DC) activation, Ag presentation, and T cell effector function has not been fully characterized. Because RA works primarily through RA receptor (RAR)α, we examined mice with a myeloid cell–specific defect in RA signaling. These transgenic mice have a CD11c-cre–driven expression of a truncated form of RARα that specifically blocks the signaling of all forms of RARs in myeloid cells. This defect results in abnormal DC function, with impaired DC maturation and activation, and reduced Ag uptake and processing. These DC abnormalities were associated with a reduced ability to mount Ag-specific T cell responses to immunization despite having normally functioning T cells. In contrast, the loss of DC-specific RA signaling did not significantly alter levels of Ag-specific Abs postimmunization and resulted in an increase in bronchial IgA. Our findings indicate that RA signaling in DCs is crucial for immune activation, and its absence impairs the development of Ag-specific effector functions of T cell immunity.

## Introduction

Vitamin A is essential for embryonic development and the maturation of the immune system (1, 2). Retinaldehyde dehydrogenase is the rate-limiting enzyme that converts vitamin A into retinoic acid (RA) derivatives, the active moieties of the vitamin (3). The most abundant vitamin A metabolite, all-*trans* RA, binds to RA receptor (RAR)α to modulate many immune-regulatory genes, leading to a variety of immune activities (4). These include increased expression of gut-homing integrins α_4_β_7_ and CCR9 on lymphocytes (5–7). In addition, RA synergizes with TGF-β to promote regulatory T cell (Treg) expansion and suppress Th9 and Th17 differentiation (8, 9). RA also supports the development of mucosal immunity by promoting effector T cell responses to infection and vaccination (10, 11). Given this range of activities, RA appears to be central to the induction of systemic and mucosal immunity and tolerance (12–14).

Dendritic cells (DCs) are crucial to initiating immune responses, and this involves DC maturation and activation to efficiently mediate Ag uptake and processing. It is thought that DC activation is partially mediated through RA as it regulates DC function in an autocrine manner and has effects on other immune cells in mucosal tissue (15). DCs initiate Th cell subset differentiation by Ag presentation (16). In the spleen, distinct myeloid DC populations CD11c^+^MHC class II (MHC II)^+^CD11b^+^ (CD11b^+^ DCs) or CD11c^+^MHC II^+^CD8α^+^ (CD8α^+^ DCs) express IRF4, IRF8, and BATF3 transcription factors (17). These lineages have distinct functional differences in eliciting Th1, Th2, or Th17 T cell responses (18–21). RA signaling is reportedly involved in the development of conventional type 1 DC (DC1; CD8α^+^ DC) and conventional type 2 DC (DC2; CD11b^+^ DC) subsets by altering their transcriptional activity (22). The role of the RA and the RARα axis in creating subsets of DCs that elicit subsets of DCs that influence T cell responses and polarization, T cell response polarization is therefore important to study.

Most studies examining the role of RA in immune function have involved either pharmacological inhibitors of RA signaling or vitamin A–deficient diets (22, 23). These approaches result in systemic vitamin A deficiency, which affects all types of immune cells. To focus specifically on RAR signaling in DCs, we used transgenic mice with a CD11c-cre–driven expression of a truncated form of RARα (RAR403) that specifically blocks the signaling of all forms of RAR in CD11c^+^ cells (24, 25). The function of the immune system of these animals was first studied in vitro and showed a specific DC defect while documenting normal lymphocytic function. We then immunized these animals and examined differences in DCs, T cell immunity, and humoral immunity compared with wild-type (WT) animals. These studies indicated that defective RARα signaling impairs DC maturation, reduces expression of DC surface costimulatory markers, and markedly decreases DC cytokine secretion. Furthermore, DCs with defective RARα signaling do not present Ags effectively and are associated with reduced capacity to activate T cells for cytotoxic responses. In contrast, inhibiting RARα signaling did not appear to alter the magnitude or type of Ab response. These results provide evidence that inhibiting RA signaling in DCs impairs T cell immunity.

## Materials and Methods

### Ag and adjuvants

Nanoemulsion (NE) adjuvant was produced by high-speed emulsification of ultra-pure soybean oil with cetylpyridinium chloride, Tween 80, and ethanol in water, with resultant NE droplets with an average diameter of 350–400 nm (26, 27). Endotoxin-free OVA was purchased from Hyglos. The endotoxin content of all vaccine components was determined by a *Limulus* amebocyte lysate assay (Pierce).

### Mice

Dominant-negative RARα (dnRARα) mice were created by cross-breeding C57BL/6J-Tg(Itgax-cre,-EGFP)4097Ach/J (stock no. 007567, The Jackson Laboratory) and ROSA26-RAR dominant-negative mice to generate CD11c-dnRARα (dnRARα) mice (25). OT-II B6.Cg-Tg(TcraTcrb)425Cbn/J animals (stock no. 004194, The Jackson Laboratory) were provided by Dr. Chang Kim’s laboratory. Animals were maintained in specific pathogen-free conditions. Mice were immunized with 6 μl per nare under anesthesia (isoflurane) for a total of 12 μl of NE (10 μg of OVA in 20% NE). PBS with OVA served as a control. Subsequent booster immunizations were performed 4 and 8 wk after the first immunization. Mice were euthanized 3 and 10 d after the last immunization. Blood was collected by heart puncture, and serum was separated by centrifugation. Bronchoalveolar lavage (BAL) was performed with 0.8 ml of PBS containing protease inhibitors. Spleens were harvested at the time of euthanasia.

Single-cell suspensions of splenocytes were made by mechanically disrupting the tissue. Cells were collected in RPMI 1640 and washed twice. RBCs were lysed with ACK (ammonium-chloride-potassium) lysis buffer and washed twice. Final cell suspensions were made in complete T cell medium (RPMI 1640 containing 10% FBS, 1× nonessential amino acids, 50 μM 2-ME, 1 mM sodium pyruvate, 100 IU of penicillin, and 100 μg/ml streptomycin), and cell suspensions were passed through a 0.7-μm filter to isolate single cells.

### Flow cytometry

The following Abs were used for staining: CD16/32 (19), CD11c-AF647 (N418), CD11b-allophycocyanin-Cy7 (M1/70), CD8α (53-6.7), MHC II-PE-Cy7 (M5/114.15.2), CD40-PE, CD80-SB600 (16-10A1), CD86-FITC (GL-1), CD4-PE (GK1.5), CD44-allophycocyanin-Cy7 (IL7), CD62L-PerCp-Cy5.5 (MEL-14), CD69-PE-Cy7 (H1.2F3), and Foxp3 (150D). All Abs were purchased from eBioscience, BD Pharmingen, or BioLegend. For surface markers staining, cells were incubated with CD16/32 Ab for 10 min to block Fc receptors. Then, cells were suspended in FACS buffer (PBS 0.1% BSA) containing Abs and stained on ice for 20 min. Cells were washed with FACS buffer and events were acquired on a NovoCyte 3000 flow cytometer (ACEA Biosciences). For Foxp3 staining, a Foxp3/transcription factor staining buffer set from eBioscience was used per the manufacturer’s instructions. Data were analyzed using FlowJo software (v10). Gating strategies for DC activation, Tregs, and T cell activation are described in Supplemental Fig. 1.

### Bone marrow–derived DC cultures

Bone marrow was harvested as previously described (27). Bone marrow–derived DCs (BMDCs) were cultured for 10 d in RPMI 1640 containing 10% heat-inactivated FBS, 1× nonessential amino acids, 50 μM 2-ME, 1 mM sodium pyruvate, 20 ng/ml GM-CSF, 100 IU of penicillin, and 100 μg/ml streptomycin. Bone marrow cells were counted and 2 × 10^7^ cells were seeded in 20 ml of complete medium in a T-150 flask. Then, 10 ml of fresh medium was added on day 3 of culture and hemidepletion was performed on days 5, 7, and 9. On day 10, BMDCs were analyzed for maturity by determining the cell surface expression of CD11c^+^CD11b^+^ cells. More than 95% of cells were double positive for CD11c and CD11b. All of the experiments were performed using a complete growth medium containing charcoal-stripped FBS (10%).

### T cell isolation

Spleens were harvested and a single-cell suspension was made as previously described. T cells were further isolated using an EasySep mouse CD4^+^ T cell isolation kit (STEMCELL Technologies, catalog no. 19852) according to the manufacturer’s protocol. Isolated cells were resuspended in T cell media.

### DC activation

BMDCs (5 × 10^5^) were seeded per well in a 96-well plate and stimulated with LPS (100 ng/ml). BMDCs were incubated for 8 h to study the signature mRNA. To quantify secreted cytokines, 1 × 10^6^ BMDCs were stimulated with LPS (100 ng/ml) for 24 h. IL-6 (R&D Systems, catalog no. IL6-DY406) and IL-12p70 (Invitrogen, catalog no. BMS6004) releases were quantified using ELISA according to the manufacturer’s protocols. Surface expression of costimulatory molecules (CD40, CD80, CD86, and MHC II) was measured through flow cytometry after a 24-h stimulation with LPS (100 ng/ml).

### Quantitative RT-PCR

RNA was isolated from BMDCs using a Direct-zol RNA MiniPrep Plus kit (Zymo Research) according to the manufacturer’s protocol. Isolated RNA was quantified using NanoDrop, and cDNA was synthesized with a high-capacity cDNA reverse transcription kit (Applied Biosystems). Power SYBR Green PCR master mix (Applied Biosystems) was used for quantitative RT-PCR. Gene expression was calculated by ΔΔCt analysis and normalized to β-actin levels. Fold change was calculated over unstimulated WT samples. Primers were purchased from Integrated DNA Technologies. Primer sequences are provided in Supplemental Table I.

### Ag uptake

BMDCs (2.5 × 10^5^) were seeded per well in a 96-well plate with 0.5–2 μg/ml OVA-AF88 (Invitrogen, O34781) and cultured for 4 h. Cells were washed with FACS buffer and stained with live/dead dye, CD11c, and CD11b. Ag uptake was measured through flow cytometry as fluorescence intensity of labeled Ag in gated CD11c^+^CD11b^+^ DCs.

### DC/T cell coculture

BMDCs (2.5 × 10^4^) were seeded per well in a 96-well plate in 100 μl of complete media with OVA MHC II peptide (InvivoGen, OVA_323–339_ at 1 μg/ml) or control peptide. BMDCs were incubated for 4 h at 37°C. The excess peptide was then washed off with FACS buffer and incubated for 24 h with T cells (2 × 10^5^) isolated from OT-II mice. T cell activation was determined by the expression of CD44, CD62L, and CD69 on gated live CD4^+^ T cells. For specific DC subset sorting, a single-cell suspension was prepared as described under *Mice* section. Cells were stained for live/dead in combination with CD11c, MHC II, CD8α, and CD11b. Two DC subsets sorted were defined as live CD11c^+^MHC II^+^CD8α^+^ (CD8α^+^ DCs) and CD11c^+^MHC II^+^CD11b^+^ (CD11b^+^ DCs). The gating strategy used for DC subsets sorting is presented in Supplemental Fig. 1D.

### Direct T cell activation

T cells were isolated from dnRARα and littermate control animals and placed on plates coated with mouse anti-CD3 (1 μg/ml) overnight. For T cell activation, 2 × 10^5^ cells were cultured for 5 h in the presence of anti-CD28 (2.5 μg/ml). T cell activation was measured as described earlier.

### Analysis of secreted cytokines in cell culture

T cell–secreted cytokines were measured from the culture supernatant after 3 d of coculture with OVA MHC II peptide–pulsed BMDCs using the Luminex multiplex detection system (Millipore, Billerica, MA).

### Measurement of serum and mucosal Abs

Serum Ig levels were measured using ELISA. High-binding plates were coated with 100 µl of OVA (20 μg/ml) in sodium bicarbonate buffer and incubated overnight at 4°C. The plates were then washed three times with ELISA wash buffer (PBS + 0.05% Tween 20) and blocked with PBS containing 1% nonfat dry milk for 1 h at 37°C. The blocking solution was removed, and diluted serum samples were added to individual wells and incubated overnight at 4°C. The plates were then washed three times and incubated with alkaline phosphatase–conjugated secondary Ab. Endpoint titers were determined by calculating cutoff OD values from the naive sera controls. The cutoff OD was determined as the mean OD (control samples) + 2 × SD (control samples). For OVA-specific IgA estimation, an anti-mouse OVA-IgA assay was used (Chondrex, catalog no. 3018) per the manufacturer’s directions.

### Statistical analysis

Data were analyzed using GraphPad Prism. Results presented are representative of at least two independent experiments. Data are expressed as mean ± SEM. The Mann–Whitney *t* test was used to compare the two groups. In experiments comparing multiple groups, statistical differences were calculated by using the Holm–Sidak/Tukey method for two-way ANOVA. Statistical significance is indicated as follows: *p < 0.05, **p < 0.005, ***p < 0.0005, and ^****^p < 0.0001.

## Results

### RA signaling in DCs facilitates Ag uptake and response to TLR-4–mediated activation

**FIGURE 1.**
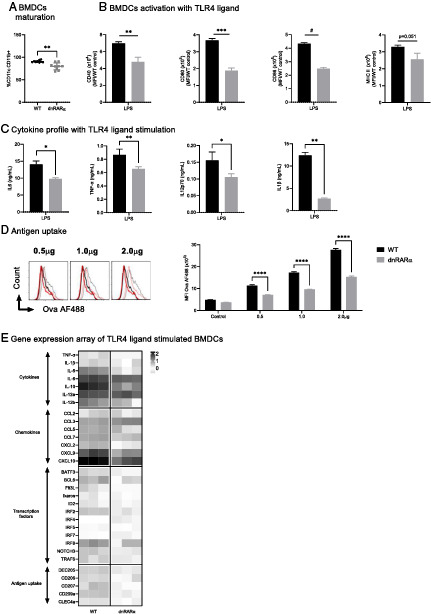
RARα signaling in DCs regulates their activation and functions. (**A**) Maturation of DCs cultured for 10 d from bone marrow cells as described in *Materials and Methods* (*n* = 3, performed in triplicates). (**B**) Cell surface expression of CD80, CD86, MHC II, and CD40 on CD11c^+^CD11b^+^ BMDCs after 24 h of in vitro stimulation with LPS (100 ng/ml). Bar graphs show mean fluorescence intensity (MFI). Changes in expression were normalized by subtracting the WT untreated control. (**C**) Cytokines were detected in the culture supernatant of BMDCs stimulated with LPS (100 ng/ml) for 24 h. (**D**) Ag uptake by BMDCs. Cells were incubated with increasing concentrations of OVA-AF488 for 4 h. Cells were washed and AF488 was read on CD11c^+^CD11b^+^ BMDCs. Left panel, Mean representative histogram. Data are representative of two independent experiments (*n* = 3). (**E**) BMDC gene expression profile with LPS and OVA stimulation. BMDCs matured for 10 d were stimulated with LPS (100 ng/ml) and OVA (1 µg/ml) for 8 h. RNA was extracted and a gene expression array was performed. Gene expression was normalized against RPS18, and fold changes were calculated over WT untreated BMDCs. All data presented are representative of two independent experiments. *p < 0.05, **p < 0.005, ***p < 0.0005, ^****^p < 0.0001.

DCs induce CD4^+^ T cell activation and polarization through the presentation of Ag with MHC II, the expression of costimulatory molecules, and the secretion of cytokines. Because RA regulates bone marrow DC1 and DC2 development, we investigated in vitro whether ablation of RA signaling would impair the effector functions of DCs. Defective RA signaling significantly reduced BMDC development as shown by the expression of CD11c and CD11b on day 10 of culture (Fig. 1A). In addition, BMDCs cultured from dnRARα animals showed a marked reduction in the expression of costimulatory molecules MHC II, CD40, CD80, and CD86 following LPS stimulation (Fig. 1B). dnRARα BMDCs also secreted significantly less IL-6, TNF-α, IL-12p70, and IL-1β (Fig. 1C). Loss of RA signaling also resulted in significantly reduced uptake of fluorescently labeled OVA Ag (Fig. 1D), and subsequent Ag processing, measured by the quenching of DQ-OVA after proteolytic cleavage, was also significantly reduced (data not shown). dnRARα BMDCs were also exposed in vitro to OVA Ag in combination with LPS and examined for the expression of LPS-induced cytokines, chemokines, transcription factors, and Ag-processing genes. Essentially all TLR-4–induced activation pathways were significantly reduced in cultured dnRARα BMDCs as compared with controls (Fig. 1E). Taken together, these data show RA signaling in DCs regulates their Ag uptake and response to TLR-4–mediated activation.

### dnRARα DCs have a reduced capacity to activate T cells

**FIGURE 2.**
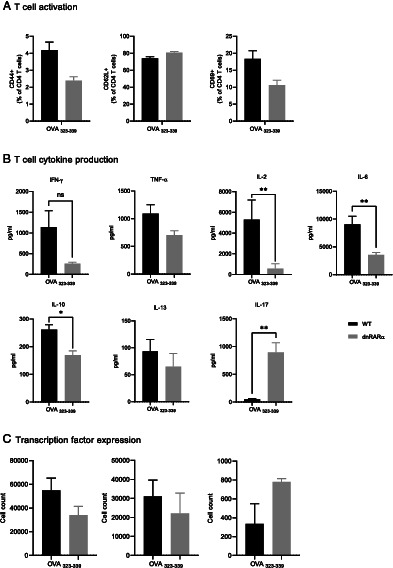
CD4 T cell activation following coculture with BMDCs. (**A**) Cell surface expression of activation markers (CD44, CD62L, and CD69) on MHC syngeneic CD4 T cells (OT-II) cocultured with MHC II peptide (OVA_323–339_)–stimulated BMDCs for 24 h. The bar graph shows the percent activated CD4^+^CD69^+^, CD4^+^CD44^+^, and CD4^+^CD62L^+^ cells. IL-12 production by CD4 T cells cocultured with OVA MHC II peptide–pulsed BMDCs is shown. (**B**) Cytokines in the culture supernatant of OVA MHC II peptide–pulsed BMDCs cocultured with OT-II CD4 T cells. (**C**) T-bet, GATA3, and ROR-γt staining of cocultured OT-II CD4^+^ T cells. All data are representative of two independent experiments. *p < 0.05, **p < 0.005.

To investigate whether dnRARα in DCs alters T cell responses, mature dnRARα BMDCs were pulsed with the MHC II peptide OVA_323–339_ and incubated in vitro with MHC syngeneic OT-II CD4^+^ T cells that specifically recognize this peptide. As compared with controls, T cells cultured with peptide-pulsed dnRARα BMDCs had reduced expression of activation markers CD44 and CD69 while retaining the expression of the naive T cell marker CD62L (Fig. 2A). The reduced expression of T cell activation markers also correlated with significantly decreased production of IL-12 and other T cell effector cytokines including IFN-γ, TNF-α, IL-6, IL-13, and IL-10, whereas IL-17 secretion was enhanced (Fig. 2B). In addition to the cytokine data, staining for transcription factors for Th1, Th2, and Th17 populations showed decreased T-bet and GATA-3 whereas RA-related orphan receptor γt (ROR-γt) was increased in T cells cocultured with dnRARα BMDCs as compared with the controls (Fig. 2C).

**FIGURE 3.**
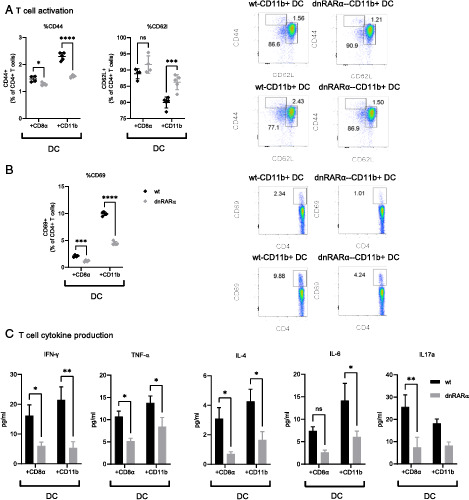
CD4 T cell activation cocultured with dnRARα DC subsets. (**A** and **B**) Cell surface expression of activation markers (CD44, CD62L, and CD69) on MHC syngeneic CD4^+^ T cells (OT-II) cocultured with MHC II peptide (OVA_323–339_)–stimulated CD11c^+^MHC II^+^CD8α^+^ and CD11c^+^MHC II^+^CD11b^+^ DC subsets after 24 h. The bar graph shows the percent activated CD4^+^CD44^+^, CD4^+^CD62L^+^, and CD4^+^CD69^+^ T cells (on left). Representative flow diagrams are shown (on the right). (**C**) Cytokines in the culture supernatant of OVA MHC II peptide–pulsed DCs subsets cocultured with OT-II CD4 T cells. All data are representative of two independent experiments. *p < 0.05, **p < 0.005, ***p < 0.0005, ^****^p < 0.0001.

Bone marrow–derived cultures result in heterogeneous DCs populations. To validate our findings in various DC subsets, we sorted CD11c^+^MHC II^+^CD8α^+^ and CD11c^+^MHC II^+^CD11b^+^ DC subsets from dnRARα and WT animals. DCs were stimulated with OVA_323–339_ peptide and cocultured with syngeneic OT-II CD4^+^ T cells. T cells cultured with CD8α^+^/CD11b^+^ DC subsets from dnRARα mice had reduced activation as shown by CD44 and CD69 expression on CD4^+^ T cells and increased expression of CD62L (Fig. 3A, 3B). Additionally, T cells cultured with dnRARα DC subsets had reduced cytokine production (IFN-γ, TNF-α, IL-4, IL-6, and IL-17a) (Fig. 3C). These data confirmed that defective RA signaling in DCs reduced T cell activation and effector cytokine production.

**FIGURE 4.**
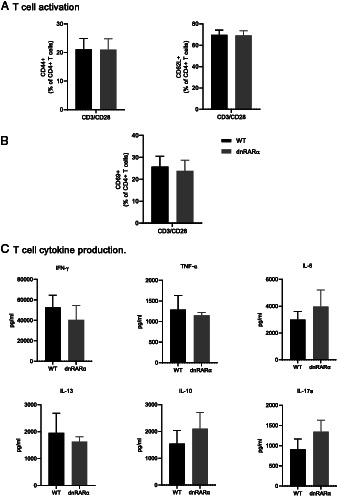
Direct CD4 T cell activation with anti-CD3/CD28. (**A** and **B**) Cell surface expression of activation markers (CD44, CD62L, and CD69) after direct CD4 T cell stimulation with anti-CD3 and anti-CD28 for 6 h from dnRARα and WT control animals. Data are representative of two independent experiments (*n* = 3). (**C**) Cytokines measured in the culture supernatant of anti-CD3/CD28–stimulated T cells after a 24-h incubation. Pooled data of two independent experiments performed in triplicates are shown.

Although T cells from the dnRARα mice have intact RA signaling, we wanted to validate normal T cell function in these mice. Isolated T cells from dnRARα animals were stimulated with anti-CD3/CD28 and compared with T cells from WT syngeneic nontransgenic mice. T cells from dnRARα animals responded similarly to those from WT animals with similar increases in CD44/CD69 activation markers as well as reductions in the naive T cell marker CD62L (Fig. 4A, 4B). Cytokines produced from dnRARα animals’ activated T cells were also not different from control animals (Fig. 4C). Therefore, the diminished immune responses in dnRARα animals were not due to impaired function of T cells.

### Inhibition of RA signaling reduces DC activation and alters splenic DC subsets

**FIGURE 5.**
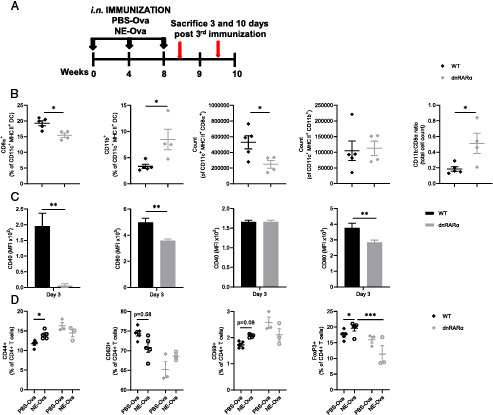
Defective DC-specific RA signaling alters DC functions. (**A**) Schedule of intranasal (i.n.) immunization with NE-OVA and PBS-OVA in WT and dnRARα mice. (**B**) DC1 and DC2 populations obtained from the spleens of dnRARα and WT littermate controls 3 d after the last immunization. (**C**) Expression of CD40 and CD80 on splenic DC1 and DC2 populations 3 d after the last NE-OVA immunization. (**D**) T cell activation in NE-OVA–immunized animals. Briefly, animals were immunized i.n. with NE-OVA and PBS-OVA. Ten days after the last immunization, splenocytes were isolated and stimulated with OVA for 24 h. Cells were stained for CD4 in combination with activation markers CD44 and CD69 as well as naive T cell marker CD62L. Isolated splenocytes are shown from NE-OVA–immunized animals stained for live CD4 and Foxp3 to identify Tregs. The gating strategy for all datasets is shown in Supplemental Fig. 1A–1C. Data are mean ± SEM (WT, *n* = 5; dnRARα, *n* = 3–4). *p < 0.05, **p < 0.005, ***p < 0.0005.

To further study the role of RA signaling in DCs in vivo, dnRARα animals were immunized with OVA using a mucosal adjuvant (NE-OVA) that activates DCs via TLR-2 and TLR-4. Splenocytes from immunized animals were harvested and stained to identify DC populations and activation markers (Fig. 5A). dnRARα animals had significantly reduced overall numbers of CD11c^+^MHC II^+^ DC (CD8α^+^ DC1s and CD11b^+^ DC2s) populations present in spleen, with DC1s markedly reduced whereas DC2s were unchanged, altering the DC1/DC2 ratio (Fig. 5B). Analysis of activation markers (CD40 and CD80) on DC subsets after NE-OVA immunization showed that dnRARα mice had significantly reduced expression of CD40 and CD80 in the DC1 population and significantly reduced CD80 expression in DC2s as compared with the WT controls (Fig. 5C). In contrast, CD40 expression in DC2s was not reduced in dnRARα animals versus WT controls.

### RA signaling is required for in vivo T cell effector functions

Splenocytes were harvested from immunized dnRARα and WT mice after immunization and were stimulated with OVA ex vivo. Activated T cells, identified as CD4^+^CD44^+^ and CD4^+^CD69^+^ cells, were increased in the NE-OVA–immunized group compared with WT animals; however, there was no enhancement of these markers on T cells from immunized dnRARα animals (Fig. 5D). Conversely, CD62L, a marker of naive T cells, was reduced in WT animals after NE-OVA immunization but was unchanged in immunized dnRARα animals (Fig. 5D). Of interest, although dnRARα animals did not show an increase in the OVA-specific CD4 T cell activation response following NE-OVA immunization, dnRARα animals had increased basal levels of activated CD4 T cells.

RA produced by CD103^+^ DCs has been reported as a regulator of Treg expansion (28). NE-OVA immunization has been shown to increase CD4^+^Foxp3^+^ Tregs in immunized animals (27, 29). Although Tregs were increased significantly after immunization in WT animals, there was no change in Tregs in dnRARα mice (Fig. 5D). Thus, these findings suggest that RA signaling in DCs regulates their activation and controls the effector functions of T cells in immunized animals.

### Alterations in Ag-specific T cell and Ab responses in dnRARα animals

**FIGURE 6.**
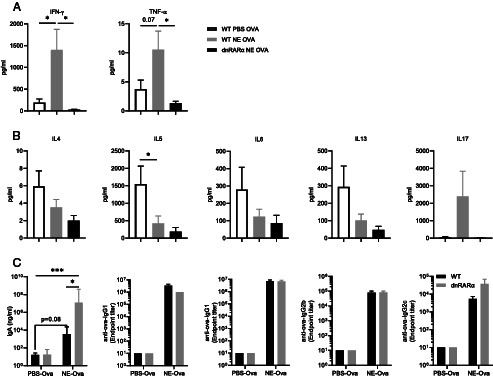
DC-specific defect in RARα signaling impairs T cell immunity without affecting humoral response in NE-OVA–immunized animals. (**A** and **B**) Th1, Th2, and Th17 cytokines from NE-OVA–immunized animals. As described earlier, animals were immunized i.n. with three administrations of NE-OVA or PBS-OVA administered at 4-wk intervals. At 10 d after the final immunization splenocytes were harvested and stimulated with OVA for 3 d and secreted cytokines were measured in cell culture supernatants. Data were normalized by subtracting cytokine levels from unstimulated samples for each animal. (**C**) OVA-specific BAL IgA, serum total IgG, IgG1, IgG2b, and IgG2c in immunized animals. Data are representative of two independent experiments (*n* = 5). *p < 0.05, ***p < 0.0005.

Immunization with OVA in NE adjuvant has been documented to skew the immune response toward a Th1/Th17 bias, increasing production of TNF-α, IFN-γ, and IL-17 while suppressing the Th2 responses with decreased IL-4, IL-5, and IL-13 production (27, 30). Despite this, dnRARα animals immunized with OVA in this adjuvant failed to produce any Th cell effector cytokines (Fig. 6A, 6B). This suggested that the loss of RA signaling in DCs markedly reduces T cell responses in vivo following immunization.

Given the reduced capacity to elicit adaptive T cell responses in dnRARα mice, we examined whether adaptive humoral immunity was compromised in these NE-OVA–immunized animals. Of interest, there were no differences in OVA-specific serum IgG in dnRARα animals when compared with WT controls (Fig. 6C). The titers of OVA-specific IgG subclasses IgG1, IgG2b, and IgG2c were comparable to control animals and, surprisingly, dnRARα animals had significantly increased OVA-specific IgA in the BAL (Fig. 6C). These data suggest that a defect in RA signaling in DCs affects T cell functions in these animals, but humoral immunity is preserved.

## Discussion

Several studies have established a role for RA in DC development (22, 31, 32); however, the exact effects of RA signaling on DC maturation and the induction of T cell effector functions remain unclear. Most prior studies of vitamin A deficiency have relied on dietary restriction or total genetic knockouts of retinol transport proteins that result in systemic RA deficiency (33–36). In this study we specifically examined the role of RA signaling in myeloid cells, focusing on DC maturation, effector function, and the ability of these cells to present Ags to generate specific T cell responses. Monocytes, macrophages, granulocytes, and different subsets of DCs express a high level of CD11c, whereas it can be expressed at low levels by neutrophils, B cells, T cells, and NK cells (37). Although CD11c is expressed variably by different immune cells, it is a classical marker to identify different DC subsets and has been used extensively to study DC functions (17, 22, 38). Using mice expressing a dominant-negative form of the RARα under the CD11c promoter, we show that defective RARα signaling in myeloid cells specifically alters the generation and activation of DCs and impairs the ability of these cells to express costimulatory molecules, secrete cytokines, and process Ag. These functional defects result in diminished Ag-specific T cell immunity despite having normal T cells. Despite this, Ab responses are still generated in immunized dnRARα mice, suggesting a unique and crucial role for RA signaling in DCs in the generation of cellular immunity.

RA signaling in DCs has been shown to control their transcriptional programming and gut homing, thereby altering DC2/DC1 ratios in the spleen and intestinal mucosa (22, 32). We, therefore, sought to determine whether these activities were specifically due to RAR signaling in myeloid cells. We studied in vitro LPS-mediated TLR-4 activation of dnRARα DCs including maturation and activation of these cells as well as Ag presentation using chromophore-conjugated OVA. Our data showed that DCs (CD11c^+^CD11b^+^) derived from bone marrow cells of dnRARα animals have significantly attenuated maturation. Also, in vitro LPS stimulation of dnRARα BMDCs resulted in significantly reduced surface expression of costimulatory molecules (CD80, CD86, and CD40) and reduced production of polarizing cytokines (IL-6, TNF-α, IL-1β, and IL-12p70). We also determine that these animals had altered ratios of splenic DC2s/DC1s, suggesting that this change was due to intrinsic DC RA signaling.

The mechanisms that appeared to underlie these defects were interesting. In a previous study, it was shown that retinoids, including all-*trans* RA, 9-*cis* RA, and retinol, regulate survival and Ag uptake of immature DCs (39). Similarly, in our examination of DCs developed from dnRARα animals, we found reduced Ag uptake and processing. This could be attributed simply to reduced Ag uptake and therefore may not be related to altered Ag processing. In addition to the dnRARα-related reduction in DC activation and Ag uptake, we showed that these animals had a decrease in the ability to induce Th1 cytokines from activated T cells. This appeared to be the result of the DC abnormalities because the T cell function in the dnRARα was found to be normal after direct stimulation with CD3/CD28, whereas dnRARα DCs were unable to activate these markers in MHC syngeneic OT-II T cells.

Further evaluation of the abnormal T cell activation in these dnRARα animals revealed reduced induction of transcription factors such as T-bet and GATA-3; however, there was an increase in IL-17 as well ROR-γt. The basis of this dichotomy is unclear; however, it has been reported that DC-derived RA inhibits transition to Th17 while also acting synergistically with TGF-β to enhance Treg generation (13).

To expand these studies and determine whether these findings are seen with in vivo stimulation, we immunized dnRARα animals with OVA using the Th1-specific mucosal adjuvant NE. NE adjuvant has been shown to increase Ag-specific Th1/Th17 immunity while suppressing Th2 in immunized animals while also increasing Tregs (27, 29, 30). Data from these dnRARα immunized animals showed a lack of T cell activation and Th1 cytokine response that was the result of defective DC activity. NE normally loads Ag to the epithelium, where it is then transferred to the mucosal DC population (40). It activates TLR-2, TLR-4, and RA pathways on DCs that enable DC activation upon immunization and results in Th1/Th17 immune responses, mucosal homing of T cells, and IgA production (27, 41). Thus, disrupting these activities would be important to NE adjuvant effects.

The replication of the in vitro defects in the in vivo studies with NE-OVA immunization likely relates to the use of a mucosal route of immunization given the gut tropism of DC2s (CD11b^−^CD8α^+^). This tropism is controlled by RA and regulates Th1 immunity through IL-12 production (32, 42). We also believed that the number of Tregs in the spleen would be decreased because of the defective RA signaling in DCs, and this was confirmed when we found that dnRARα animals had reduced Tregs following NE-OVA immunization. This decrease in Treg numbers and activity is consistent with studies that have demonstrated an increased efficacy of DC tumor vaccines by blocking RARα signaling and reducing Treg development (43). The lack of RA signaling in DCs appears to alter T cell immunity in several ways.

Interestingly, our data show two unexpected findings. First, we found that normal Ab responses were still generated in these animals, with dnRARα animals having elevated IgA production following immunization. This finding is different from what is observed in mice depleted of RAR via a vitamin A–deficient diet. Those animals lack IgA-secreting plasmacytoid cells in the small intestine, and RA instructs Ig class switching to IgA in B cells (44, 45). Second, it appeared that when RARα signaling deficiency is restricted to DCs, the production of the inflammatory cytokines is decreased. This differs from the results of Manicassamy et al. (46) who showed that RA produced by zymosan-treated DCs can act in an autocrine manner to suppress the expression of proinflammatory cytokines, namely IL-6, TNF-α, and IL-12. The difference in results may indicate that limiting RARα signaling defects to DCs may allow some immune function.

In summary, our data document that myeloid cell RARα signaling plays an important role in regulating DC maturation, cytokine secretion, and activation. Our findings confirm the importance of RA in DC development and function but also show that dysregulated RA signaling in DCs leads to attenuated T cell immunity, especially for effective immunization responses.

## Supplementary Material

Supplemental 1 (PDF)
