# Supplementary material for: Retinoic Acid Signaling Is Required for Dendritic Cell Maturation and the Induction of T Cell Immunity
Source: Immunohorizons. 2023 Jun 21;7(6):480–92. doi: 10.4049/immunohorizons.2300022 (PMC10580129; doi:10.4049/immunohorizons.2300022)
Supplement: Supplemental 1 (PDF) [file IH_2300022_Supplemental_1.pdf]

	**Transcription factors**	
**IRF8**	Forward Sequence	GACCATGTTCCGTATCCCCTGGAAG
	Reverse Sequence	GGGACCGGTCAGTCACTTCTTCA
**ID2**	Forward Sequence	TCCTGTCCTTGCAGGCATCTGAAT
	Reverse Sequence	AACGTGTTCTCCTGGTGAAATGGC
**Batf3**	Forward Sequence	CAGACCCAGAAGGCTGACAAG
	Reverse Sequence	CTGCGCAGCACAGAGTTCTC
**BCL6**	Forward Sequence	CAGAGATGTGCCTCCATACTGC
	Reverse Sequence	CTCCTCAGAGAAACGGCAGTCA
**IRF2**	Forward Sequence	GCCAGGTTGTAGAAGTGACCAC
	Reverse Sequence	CTGCGTTCTCTTCATCACTGGC
**IRF4**	Forward Sequence	GAACGAGGAGAAGAGCGTCTTC
	Reverse Sequence	GTAGGAGGATCTGGCTTGTCGA
**Flt3L**	Forward Sequence	CGCTGGATAGAGCAACTGAAGAC
	Reverse Sequence	TGTTGGTCTGGACGAATCGCAG
**Notch3**	Forward Sequence	CCACCTGCAATGACTTCATCGG
	Reverse Sequence	TCGATGCAGGTGCCTCCATTCT
**TRAF6**	Forward Sequence	TTTCCCTGACGGTAAAGTGCCC
	Reverse Sequence	ACCTGGCACTTCTGGAAAGGAC
**Ikaros exon 3**	Forward Sequence	GCCGTACAAGTGTGAGTTCTGC
	Reverse Sequence	CTCGGCTTTGATGTGTCTTGCC
**Ribosomal protein 18s**	Forward Sequence	CGGAAAATAGCCTTCGCCATCAC
	Reverse Sequence	ATCACTCGCTCCACCTCATCCT
	**Antigen uptake**	
**DEC205**	Forward Sequence	TGTCCTGAAGGCTGGCACACTT
	Reverse Sequence	CTTGGCAGAACCTTTCGGCTTC
**CD206/Mrc1**	Forward Sequence	GTTCACCTGGAGTGATGGTTCTC
	Reverse Sequence	AGGACATGCCAGGGTCACCTTT
**CD207/langerin**	Forward Sequence	GAAACCTCTCTGTATCTGCGTGG
	Reverse Sequence	GGTGCTGATGTTGTCCACACGA
**CD209a/DC-SIGN**	Forward Sequence	GCACTCCATCAAAGGCTTTGGC
	Reverse Sequence	CAAACAGCTAGGAAGAGCACCTG
**CLEC4a4/DCIR**	Forward Sequence	GGCATCTTGGAATGAGAGTGAGG
	Reverse Sequence	CCAGCACTTGTGTTCAGGTTGC
**CLEAC7a/Dectin 5**	Forward Sequence	CCAGCTAGGTGCTCATCTACTG
	Reverse Sequence	CCTTCACTCTGATTGCGGGAAAG
	**Chemokine**	
**CCL2**	Forward Sequence	GCTACAAGAGGATCACCAGCAG
	Reverse Sequence	GTCTGGACCCATTCCTTCTTGG
**CCL7**	Forward Sequence	CAGAAGGATCACCAGTAGTCGG
	Reverse Sequence	ATAGCCTCCTCGACCCACTTCT
**CCL5/RANTES**	Forward Sequence	CCTGCTGCTTTGCCTACCTCTC
	Reverse Sequence	ACACACTTGGCGGTTCCTTCGA
**CXCL2**	Forward Sequence	CATCCAGAGCTTGAGTGTGACG
	Reverse Sequence	GGCTTCAGGGTCAAGGCAAACT
**CXCL9**	Forward Sequence	CCTAGTGATAAGGAATGCACGATG
	Reverse Sequence	CTAGGCAGGTTTGATCTCCGTTC
**CXCL10**	Forward Sequence	ATCATCCCTGCGAGCCTATCCT
	Reverse Sequence	GACCTTTTTTGGCTAAACGCTTTC

Supplementary Table 1: Genes primer sequences used in the study.

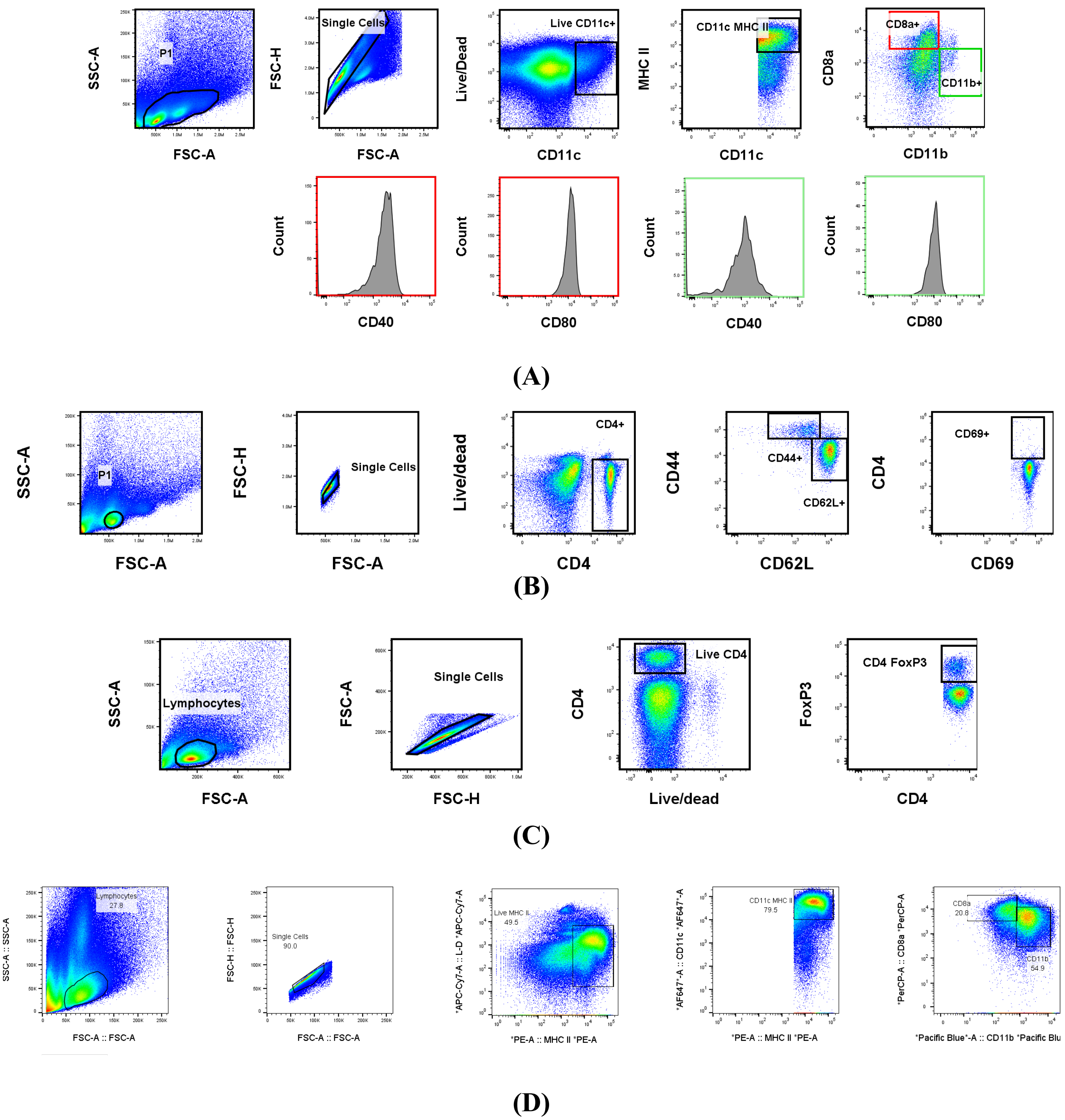

Figure S1: Gating strategy. (A) Flow cytometry gating strategy for spleen DCs shown in Figure 3A-B. (B) Gating strategy for CD4 T cells activation markers are shown in Figure 3C. (C) Gating strategy for $CD4^+$ $FoxP3^+$ T cells is depicted in Figure 3D. (D) Gating strategy for the sorting of DC subsets ($CD11c^+MHC\text{-}II^+CD8\alpha^+$ and $CD11c^+MHC\text{-}II^+CD11b^+$).
